# Experiences and perspectives on traditional and faith healers’ involvement in the care of people with severe mental health conditions in ethiopia: a scoping review

**DOI:** 10.1186/s13033-025-00691-9

**Published:** 2025-12-08

**Authors:** Mekonnen Tsehay, Teshome Shibre Kelkile, Wubalem Fekadu, Alex Cohen, Desalegn Kiros, Charlotte Hanlon

**Affiliations:** 1 Department of Psychiatry and WHO Collaborating Centre for Mental Health Research and Capacity-Building, School of Medicine, College of Health Sciences, Addis Ababa University, Addis Ababa, Ethiopia; 2 Horizon Network Zone 3, Toronto, NB Canada; 3 Department of Psychiatry, Dalhousie University, Toronto, NS Canada; 4 Department of Population Health, London School of Hygiene & Tropical Medicine, London, UK; 5 School of Nursing, Midwifery, and Health Practice, Victoria University of Wellington, Wellington, New Zealand; 6 Division of Psychiatry, Centre for Clinical Brain Sciences, University of Edinburgh, Edinburgh, Scotland, UK; 7 Centre for Innovative Drug Development and Therapeutic Trials for Africa, College of Health Sciences, Addis Ababa University, Addis Ababa, Ethiopia

**Keywords:** Traditional healers, Faith or religious healers; psychotic disorder, Bipolar disorder, Mental health, Severe mental health conditions, Serious mental illness, Africa

## Abstract

**Background:**

Traditional and faith healers (TFHs) play a prominent role in the care of people with severe mental health conditions (MHCs) in many countries. Consequently, there have been calls for closer collaboration between TFHs and mental health care practitioners. This scoping review aimed to map the literature on the experiences of, and perspectives on, traditional and faith healing for people with severe MHCs in Ethiopia.

**Methods:**

The review was conducted in accordance with the Joanna Briggs Institute methodology for scoping reviews. Pubmed, Embase, CINAHL, Scopus, Web of Science, and PsycINFO databases were searched from the earliest available records to May 2024. Online student MSc/PhD theses and catalogued Ethiopian publications up to 2015 were also searched. Studies were included if they were in English and of any study design using primary data collection. Narrative synthesis was chosen for data synthesis.

**Results:**

Of the 3,824 records identified, 31 were included. There were 17 qualitative, 12 quantitative, and two mixed methods studies, conducted in most regions in Ethiopia but with more focus on urban than rural settings. Findings were synthesised under the following themes: perceived causes of MHCs; pathways to care and help-seeking preferences; identification and intervention methods used by TFHs; experience of treatment, satisfaction with care, gaps, and barriers; and collaboration between TFHs and mental health practitioners. People with severe MHCs commonly accessed TFHs first and alongside biomedical care. A substantial range of healers was identified but they were not accessible or acceptable to all communities equally. TFH interventions were diverse and some of their practices were reported to be harmful. However, there were few in-depth studies of TFH care processes. Furthermore, there was little evidence about the experience of care from the perspective of people with severe MHCs. Efforts toward collaboration emphasised the need to develop relationships within which differences could be negotiated.

**Conclusion:**

Although much is known about the place of TFHs within care pathways for people with MHCs in Ethiopia, there are evidence gaps in relation to the perspectives of people with MHCs and rich contextual understanding of healing processes, both of which are needed for meaningful collaboration to occur.

**Supplementary Information:**

The online version contains supplementary material available at 10.1186/s13033-025-00691-9.

## Introduction

Traditional medicine, according to the World Health Organisation (WHO), is *“the knowledge*,* skills and practices based on the theories*,* beliefs and experiences indigenous to different cultures*,* used in the maintenance of health and the prevention*,* diagnosis*,* improvement*,* or treatment of physical and mental illness*” [1]. Non-biomedical approaches to healing have been referred to by a variety of names, including traditional, alternative, complementary, integrative medicine, traditional healing, or traditional and faith healing. Despite having divergent meanings around the world, these names are frequently used interchangeably, and there are no agreed-upon definitions [2]. In global mental health, this terminology of traditional and faith healing (TFH) has been used more than other terms and appears to be more inclusive, with a strong focus on the supernatural, religion, and the spirit world [3].

There has been increasing acknowledgement of the importance of TFH all over the world, including in Western countries [4–6]. Mental health conditions (MHCs) are a category of illness experience that often comes within the remit of TFH practitioners. The most commonly reported reasons for the use of TFH by people with MHCs are its cultural acceptability, perceived benefits in terms of outcomes that are valued by affected individuals, perceived relative affordability, and greater accessibility relative to scarce biomedical mental health services [7–11]. Evidence is scanty but indicates that the interventions provided by TFH practitioners can reduce distress and improve mild symptoms of common MHCs such as depression and anxiety. However, there is little evidence for impacts on the symptoms of more severe MHCs, for example, schizophrenia or other psychotic disorders [12]. Nonetheless, it is held that a shared worldview around aetiology, remedy, and expected outcome is central to the benefit of any therapy [13].

There are global imperatives to scale-up access to (biomedical) mental health care, led by the World Health Organization with its mental health Gap Action Programme (mhGAP) that aims to integrate mental health into general and primary healthcare services [14]. WHO’s Comprehensive Action Plan for mental health scale-up (2013–2030), which was endorsed by all member states, recommends collaboration with traditional and faith healers [15]. Recent trials of collaborative models between TFH practitioners and mental health care for people with severe MHCs in Nigeria and Ghana [16] and mental health care integrated into prayer camps in Ghana [17] showed that integrating biomedical care within TFH improved outcomes and was cost-effective. Such involvement of TFHs in the health system needs to draw on the strengths of both types of healing, which in turn requires an in-depth understanding of the processes, experiences, and impacts of different healing approaches.

In Ethiopia, there is a great diversity of traditional and faith healers and healing practices for a wide range of health conditions. Healers include herbalists, spiritual or faith-based healers, bone setters, those who perform surgical operations such as cauterization, bleeding, cupping, circumcision, or cutting, and sorcerers. Healing approaches are similarly diverse, from prayer, exorcism, amulets, bewitchment, animal sacrifices, fumigation, beating, and others [18–20]. Holy water sites, linked to the Ethiopian Orthodox Church, are particularly widespread and not just accessed by those who identify themselves as Orthodox Christians. Ethiopia has policies and strategies in place to encourage and promote the appropriate use and conservation of traditional medical knowledge. Research and development initiatives are seeking to validate the safety, efficacy, and quality of TFH [21–23].

The nature and amount of research about traditional and faith healing in the management of severe MHCs in Ethiopia is unknown. Based on database and grey literature searches, as well as a check of the PROSPERO international prospective register of systematic reviews, we are not aware of any previous systematic or scoping review on this topic. Therefore, the objective of this scoping review was to map the literature on the roles, perspectives, and experiences of traditional and faith healing for people with severe MHCs in Ethiopia. Severe mental health conditions (MHCs) lack a universally accepted definition and are often used interchangeably with terms such as severe and persistent, serious, disabling, or chronic mental illness. In this study, articles were included if they referred to individuals with probable or confirmed diagnoses of psychotic disorders, including schizophrenia spectrum, schizoaffective, and mood disorders with psychotic features or severe forms of depression.

The following research questions framed this review: Who is involved in TFH for people with severe MHCs in Ethiopia? How do people with severe MHCs in Ethiopia conceptualize their needs for care and healing? What are the processes of TFH care, how are they experienced and perceived, and what is their impact? How can traditional and faith healers collaborate with mental health care practitioners in the management of people with severe MHCs?

## Methods

This scoping review was conducted in accordance with the guidelines set by the Joanna Briggs Institute (JBI) [24] to ensure rigour and facilitate replicability. The findings have been reported in line with the Preferred Reporting Items for Systematic Reviews and Meta-Analyses-Scoping Review Extension (PRISMA-ScR [25]. The review protocol is registered at Open Science Framework (OSF) (DOI: 10.17605/OSF.IO/75DR8).

### Eligibility criteria

The eligibility criteria for this scoping review were structured in terms of Population, Concept, and Context (PCC) [26]. Our review included published literature pertaining to the experiences and perspectives on traditional and faith healers’ involvement in the care of people with severe MHCs in Ethiopia. In this review, we included all English language literature, reporting any type of outcome, regardless of study design. In addition, we used specific inclusion and exclusion criteria described in the table below (Table 1).

**Table 1:** Inclusion and exclusion criteria

Inclusion Criteria	• Related to traditional and faith healers and healing as defined by WHO 2013 (1) • Related to people with severe MHCs, including psychotic disorders, and severe affective disorders (e.g., bipolar disorder), assessed by clinical diagnosis or likely to have chronic/severe MHCs without a formal clinical diagnosis. • Discusses the views and/or experiences of Ethiopian traditional or faith healers in the identification and/or management of severe MHCs. • Includes primary data, regardless of its quality evaluation or study design. • Written in English.
Exclusion Criteria	• Conference abstracts. • Studies focusing on people with common MHCs, including depression and anxiety. • Studies on people with developmental disabilities unless co-morbid with a severe MHC. • Studies carried out exclusively in those under 18 years of age or in samples where the majority are under 18 years and the findings for adults cannot be extracted separately.

### Information of sources

We searched multiple databases: Pubmed, Embase (Elsevier), CINAHL with Full Text (EBSCO), Scopus (Elsevier), Web of Science (Clarivate), and PsycINFO, from the earliest available records to May 2024. We also searched University repositories for student MSc/PhD theses and used catalogued Ethiopian mental health-related publications up to 2015.

### Search strategy

**Fig. 1:**
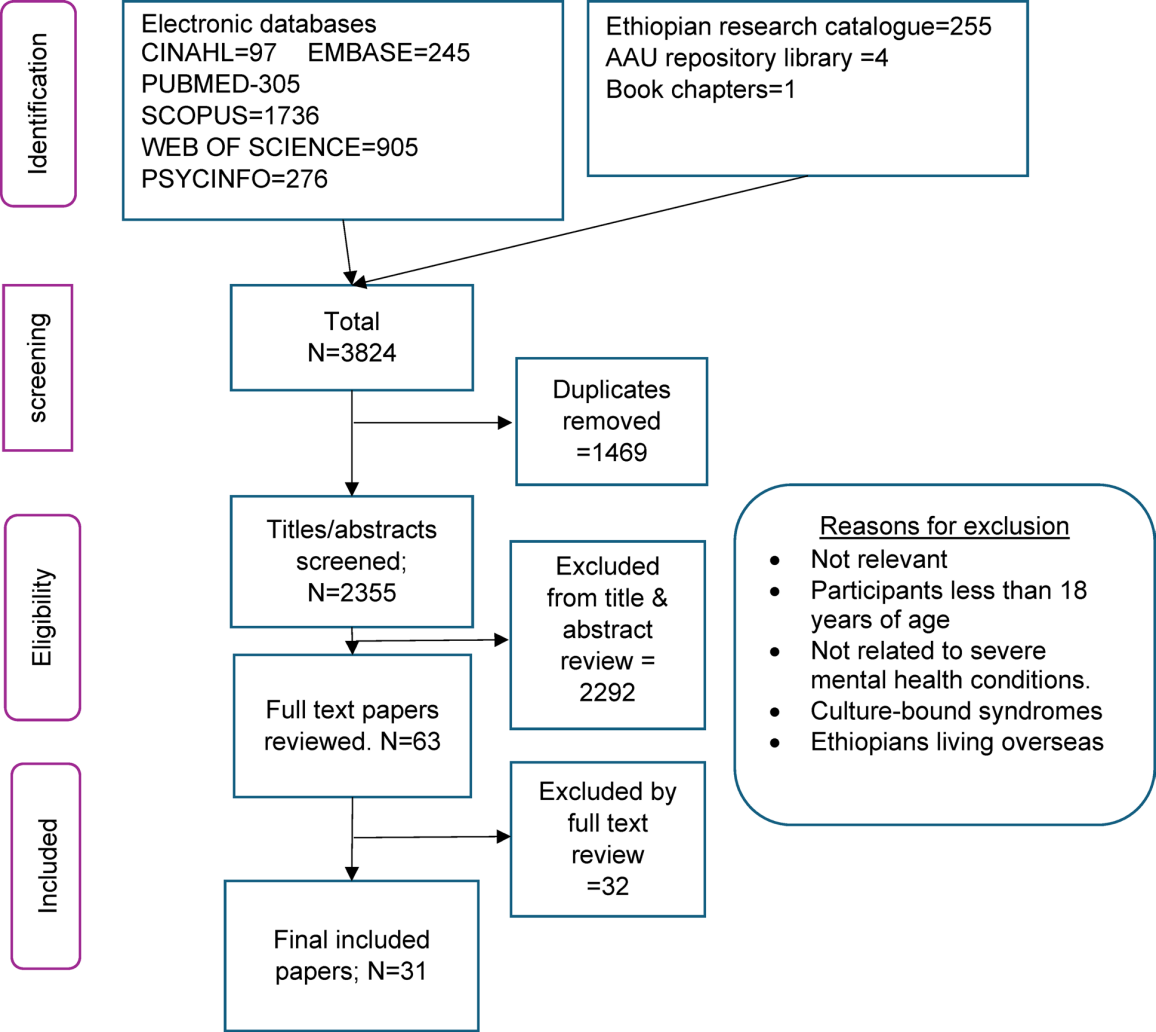
PRISMA flow chart

We conducted an initial search of Scopus and Pubmed to identify relevant keywords contained in the title, abstract, and subject descriptors. The identified terms and the synonyms were used to search the other databases, for a comprehensive search of the literature (Fig. 1).

The key terms we used for searching were: ((Traditional OR spiritual OR Folk OR indigenous OR religious OR Contemporary OR alternative OR holistic OR faith OR multifaith) AND (heal* OR medicine OR remed* OR therapy OR assistance OR intervention* OR counselling OR treatment OR assessment) AND (Mental OR psychiatric OR psychological) AND (Illness* OR disorder* OR disturbance OR problem OR impairment OR health OR conditions) AND (Ethiopia)). The detailed search strategy is in the Supplementary file, Table 3.

Reference lists and bibliographies of the identified articles were searched. After full articles were retrieved, forward and backward citation tracking was carried out to identify any further articles.

### Source of evidence selection

All identified citations were collated and uploaded into Rayyan QCRI, a computer-based application to support the conduct of systematic reviews [27], and duplicates were removed. Titles and abstracts were screened by two independent reviewers (MT and DE) against the eligibility criteria. Any disagreements between the two reviewers were resolved through discussions. Full copies of articles were obtained and assessed to see if they met the inclusion criteria. Citation tracking and review of reference lists of relevant papers were used to identify any additional papers.

### Data extraction (charting) and data items

The extraction form included author/year, location, methodological approach, participants, research questions/aims, and main themes/findings. MT charted the data. The charting was reviewed and revised in discussion with co-authors. We then classified articles based on the methodological approach, as qualitative, quantitative, or mixed methods (Supplementary file, Table 1).

### Critical appraisal of evidence

Because of the diversity of the study designs, we used the Quality Assessment for Diverse Research (QuADS) approach to quality appraisal [28]. Quality assessment was used to inform the narrative synthesis. We did not exclude any articles that had low quality based on QuADS (Supplementary file, Table 2).

## Synthesis of results

In order to capture all relevant findings, given the substantial heterogeneity of study designs and approaches, narrative synthesis was chosen as the preferred approach for data synthesis. The three steps of narrative synthesis, as outlined by Popay and colleagues, were followed: (a) develop a preliminary synthesis, (b) explore relationships in the data and (c) assess the robustness of the synthesis [29]. The evidence was presented in tabular format with themes/categories in a manner that aligned with the objective of the scoping review.

## Results

### Characteristics of included studies and search results

The initial searches identified 3824 records from the electronic databases, 255 articles from the catalogue of Ethiopian research, four papers from the repository at Addis Ababa University, and one book chapter recommended by contacted experts. The number decreased to 2355 when duplicates were removed. Based on the eligibility criteria, 2292 publications were excluded during the initial review of titles and abstracts. Full-text evaluation was carried out on 63 papers. Of these, 31 articles were eligible and included (Fig. 1).

**Fig. 2:**
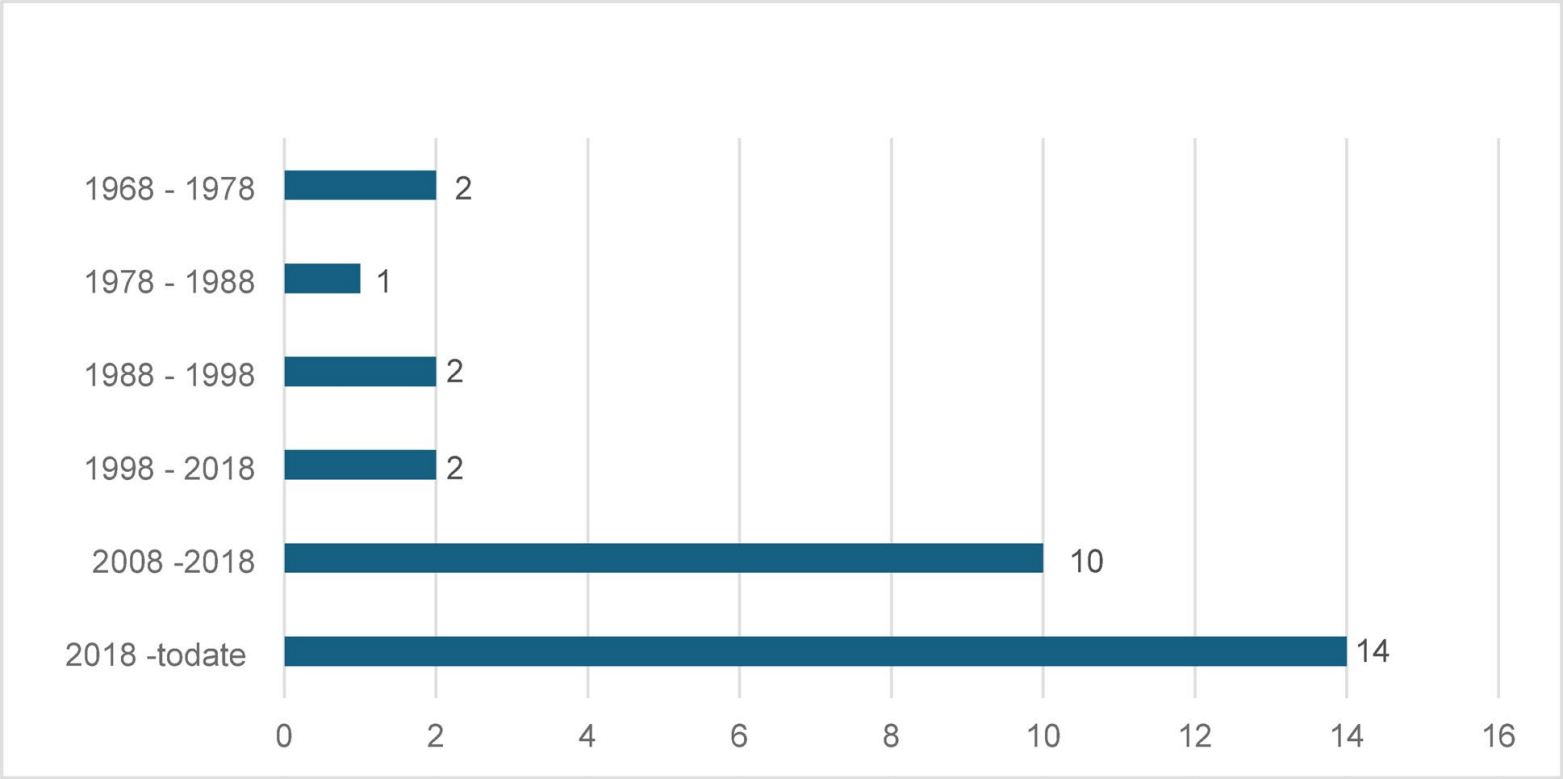
Number of publications over time

Nine studies were carried out in Ethiopia’s capital city of Addis Ababa. Ten studies were carried out in the regional towns, including Jimma, Gondar, Mekele, Arbaminch, Dessie, and Asossa. One study was undertaken in both Addis Ababa and Assela (rural). Eleven additional studies were conducted in rural locations, including Gojjam, Sodo, Shewa, Borana, Lalibela, Butajira, Waliso, and Nekemte. The studies were published between 1968 and 2024, with a notable increase in the number of papers on this topic in the last decade (Fig. 2).

Of the 31 studies, twelve were quantitative, two were mixed-method studies, and seventeen were qualitative. The qualitative studies used various data collection techniques. Six studies were ethnographic in nature or utilised observation as one of the data collection methods, and the remaining qualitative studies used key informant interviews, case vignettes, document analysis (from the web, minutes of stakeholder meetings, and religious books), informal interviews, in-depth interviews, and focus group discussions.

Three studies examined ongoing efforts to establish a collaboration between mental health services and Ethiopian Orthodox Christian holy water sites in Addis Ababa. The first study analysed clinical records of 1,888 people with MHCs over seven years from a temporary clinic adjacent to a holy water site [30]. The second study conducted in-depth interviews with 14 holy water attendants. These attendants run group houses for holy water residents and are paid for by family members. In addition, clinical records data of 174 individuals who attended the clinic in the three years since it opened were analysed descriptively [31]. The third study involved in-depth interviews with holy water healers at St Michael’s and St Mary’s churches in Entoto [32].

Of the two mixed-method studies, one study combined focus group discussions and a cross-sectional survey, using the General Help-Seeking Questionnaire (GHSQ) [33]. The second mixed methods studysimilarly used in-depth interviews and focus group interviews complemented by self-report questionnaires: the Mental Health Literacy Scale (MHLS) and the Social Support Scale (The Oslo 3-items) [34].

Of the quantitative studies, three studies used the World Health Organization Encounter form to assess help-seeking patterns and pathways [35–37]. A further two quantitative studies employed structured questionnaires, developed by the authors, to portray the profile and experience of community members and TFHs in relation to people with severe MHCs [20, 38].

The remaining seven studies used structured questionnaires, including the Alcohol, Smoking, and Substance Involvement Screening Test (ASSIST), Brief Psychiatric Rating Scale (BPRS) [39], Butajira Treatment Gap Questionnaire (TGQ) [40], Medication Adherence Rating Scale (MARS) [41], Causal Models Questionnaire for Schizophrenia (CMQS) [42], Community resource inventory (semi-structured questionnaires) [18], and General Help-seeking Behaviour questionnaire (GHSQ) [43]. The sample sizes for quantitative studies ranged from 178 (for TFHs) to 1888 (for individuals with MHCs) (Table 2).

**Table 2:** Description of included articles

Author/year	Location,	Setting	Study design	Methodological approach/measures	Participants and sample size	Research questions/aims
Alem A. et al. 1999	Butajira, rural District town	Community-based	Qualitative	Key informants (KI) interviews, using the WHO KI questionnaire and case vignettes	100 selected Community and religious leaders	Mental health help-seeking and preferred interventions
Anderson L. 2007 (preprint)	Lalibela, rural District town,	Ethiopian orthodox Christian church and holy water	Qualitative	Document analysis of articles and religious books, interviews with priests and community members and observation of holy water healing	Community members, priests, and people cured with church healing	To examine the healing practice of the Ethiopian orthodox church and its relationship with other THs
Asher, L. et al. 2021	Addis Ababa, capital city	Entoto St. Mary and St. Mikael holy water	Qualitative	Analysis of clinic records, and in-depth interviews	14 holy water attendants and service users of Entoto holy water healing	The practice of holy water healing for mental illness: holy water attendants’ role and attitudes
Bahretibeb Y et al. 2024	Addis Ababa, capital city	Entoto St. Mary and St. Mikael holy water	Qualitative	In-depth interviews	12 holy water healers of St Michael’s and St Mary’s churches	Faith healers’ views on mental illness, treatment methods, and their attitude towards collaboration
Baheretibeb, Y. et al. 2022	Addis Ababa, capital city	Entoto St. Mary and St. Mikael holy water	Qualitative	Examined clinic records, meeting minutes, workshops, and informal participant interviews.	Psychiatrists. psychiatric trainees, and faith healers from two holy water sites	Collaboration between faith healers and biomedical practitioners
Baye Berihun 2015	Addis Ababa, capital city	Entoto Kidane-Mihret holy water	Qualitative	In-depth interviews with four religious healers and two clients and observation of the treatment process	4 Entoto Kidane-Mihret Monastery priests and 2 people with mental illness	Faith as a means of healing
Desalegn A. et al. 2016(preprint)	Gondar, district town	THs clinic	Qualitative	In-depth interviews and observation	8 traditional healers (THs) and 2 their clients with mental illness	Conceptualisation of mental illness and treatment practices among traditional healers
E. Fuller Torrey 1972 (book chapter)	Waliso (Oromia)	Ghion holy water	Qualitative	Observation and informal interviews with the healer and community around.	Ghion holy water healer (Abba Wolde Tensaye)	The practice of faith healers at the holy water site
Gutema, B. T. and Mengstie, M. M.2022	Berta community in Assosa	Community-based	Qualitative	7 key informant interviews	Traditional healers	Perceptions, identification, and treatment of mental illness by THs.
Jacobsson, L. Merdasa, F. 1991	Nakamte, Oromia region	Community-based	Qualitative	In-depth interviews	Coptic priests, muslim sheikhs, and other traditional healers	Describing perceptions of mental disorders and the various types of traditional healers and treatments.
Kortmann F. 1987	Addis Ababa	Medical trainee of AAU	Qualitative	Essay writing based on guiding questions, clinical experience and literature consultation.	21 medical students who had just completed psychiatry training.	Overviews of traditional and professional mental health care in Ethiopia
Monteiro NM, Balogun SK 2013	Addis Ababa and Asella	Community- and hospital based	Qualitative	In-depth interviews	75- community members, 35 healthcare workers, and 5 traditional healers.	Traditional perceptions and treatment of mental disorders in Ethiopia
Robert Giel 1968	Waliso, Oromia region,	Ghion holy water	Qualitative	Focused ethnography: used Priest’s recordings and Observations	Holy water healers, and service users	Faith-healing and spirit-possession in Ghion, Ethiopia
Seblewengel D. et al. 2016 (thesis)	Addis Ababa, capital city	Kale Heywet and Muluwongel Believers’ Church	Qualitative	Literature consultation, in-depth interviews and observation	4 ministers, healers, 4 gifted healers; 2 Service users	Perceived causes, identification methods, and treatment practices of mental illness in evangelicals
Shibre T. et al. 2008	Butajira, district town	health care and TH clinics setting	Qualitative	Key informant interviews	24 THs, service users of THs, and patients of health centres	Traditional treatment of mental illness; perception, practice, and satisfaction
Tadesse Z. et al. 2018 (Thesis)	Addis Ababa, capital city	Holy water-based	Qualitative	Informal and in-depth interviews, observation at service sites, and review of secondary data	People at Shinkuru Mikael holy water and their family members, friends, neighbours, and faith healers	To explore the process of holy water therapy for mental disorders
Tefera S. & Shibrie T. 2012	Borena, rural community	Community-based	Qualitative	Key informants’ interviews and six focused group discussions	Borena semi-nomadic people	Perceived causes of severe mental disturbance and preferred interventions
Anbesaw T. et al. 2024	Dessie, district town	Community-based	Mixed method	-Qualitative, in-depth interviews and FGDs -Quantitative using the Mental Health Literacy Scale (MHLS) is a 35-item and Oslo Social Support 3-item questionnaire	343 traditional healers in Dessie town	Mental health literacy of traditional healers and associated factors
Yeshanew B. et al. 2020	Mertulemaryam, district town	Community-based	Mixed method	Structured interviews using a General Help-Seeking Questionnaire (GHSQ) and focus group discussions	964 community participants of Mertulemaryam town	Help-seeking for mental health problems
Baheretibeb Y. et al. 2021	Addis Ababa, capital city	Holy water-based	Quantitative	Workshops and examination of clinical records of 1888 people with MHCs over 7 years at the newly created clinic.	Attendees of St. Michael’s Church and St Mary’s church and psychiatrists and psychiatric trainees	Active collaboration between biomedical and TFHs, usage of the newly created clinic.
Bekele Y. et al. 2009	Addis Ababa, capital city	Hospital-based	Quantitative	- WHO encounter form questionnaires + sociodemographic variables.	1044 people with a new presentation to Amanuel Specialized Psychiatric Hospital	Pathway to psychiatric care
Belachew Y. et al. 2019 (preprint)	Sodo District	Community-based	Quantitative	Interviews using fully structured questionnaires prepared by authors	173 traditional healers from Sodo	Pathways to psychiatric care, experience, and intention of collaboration in Ethiopia
Boti, N. et al. 2020	Arbaminch, district town	Community-based	Quantitative	Structured interviewer-administered questionnaires prepared by authors and case vignettes	617 community members	Community perceptions and attitudes towards people with schizophrenia
Fekadu W. et al. 2015	Addis Ababa, capital city	Holy water-based	Quantitative	-WHO’s ASSIST and Brief Psychiatric Rating Scale (BPRS).	416 Entoto St. Mary holy water users	Prevalence and associated factors of mental illness among holy water users
Fekadu A. et al. 2019	Butajira, district town	Community-based	Quantitative	Interview with Butajira Treatment Gap Questionnaire (TGQ)	300 people with psychosis	Psychosis treatment gap and its consequences
Fikreyesus, M. et al. 2016	Jimma, district town	Hospital-based	Quantitative	Sociodemographic variables and Medication Adherence Rating Scale (MARS).	386 people with psychotic disorders at Jimma University hospital	relapse and association with the use of TFHs among people with psychotic disorders.
Mekonen E. G. et al. 2020	Maksegnit, district town	Community-based	Quantitative	Socio-demographic variables and Causal Models Questionnaire for Schizophrenia (CMQS)	435 Community members	Perceived causes and determinants of help-seeking behaviour for schizophrenia
Girma, E. and Tesfaye M. 2011	Jimma, a district town	Hospital-based	Quantitative	Chart reviews and interviews using structured questionnaires.	384 people with MHCs attending Jimma University Hospital	Patterns of treatment-seeking behaviour for mental illnesses
Selamu M. et al. 2015	Butajira, district town	Community-based	Quantitative	Interview using a community resource inventory by trained Health Extension Workers (HEWs)	Community mapping	To map community resources, including traditional and faith healing, available for mental health care
Tesfaye, Y. et al. 2020	Jimma Zone, Seka Chekorsa district	Community-based	Quantitative	Sociodemographic and General Help-Seeking Behaviour Questionnaire (GHSQ)	423 community members	Patterns of treatment-seeking behaviour for mental illnesses
Teshager, S. et al. 2020	Mekele, regional town	Hospital-based	Quantitative	Sociodemographic variables and the WHO Encounter Form	423 people with mental illness attending Ayder Hospital	Pathways to psychiatric care and factors associated with delayed help-seeking

### Participant characteristics

The following groups were used as sources of information or participants in the studies: community leaders, religious leaders (including Orthodox Christian priests, Muslim sheikhs, and protestant Christian ministers), traditional and faith healers, psychiatrists, psychiatric trainees, healthcare professionals, holy water users, holy water attendants who manage group houses for holy water residents paid by family members, and people with MHCs and their family/caregivers.

### Quality appraisals of the studies

According to QuADS (Supplementary file, Table 2), no significant methodological quality issues were identified. However, in the ethnographic studies, observations were limited to brief periods, with visits to healing locations lasting no more than a week [44–48]. In all studies, the lowest score was found for item 12, which assesses the evidence for considering research stakeholders in the research design or conduct. Some quantitative studies also scored lowest at item 7, which evaluates whether the format and content of the data collection tool are appropriate to address the stated research aim(s).

### Narrative synthesis

We identified five themes: perceived cause of mental health conditions, pathway to care and help-seeking preferences, identification and intervention methods used by TFHs, the experience of treatment satisfaction, gaps and barriers, and collaboration between TFHs and mental health practitioners (Table 3).

**Table 3:** Data extracted from the 31 studies based on the scoping review research questions

Theme	Sub-themes
Perceived causes of mental health conditions	Qualitative findings: • Supernatural forces like possession by evil spirits, curses, bewitchment (ghosts, malevolent spirits, sorcerers, or magicians), and punishment from God *(yefetari quta)* ADDIN EN.CITE [13, 17, 18, 20, 32–34, 36, 38, 42, 44, 48–53] • Exposure to the wind, thinking too much, excessive worry, and work stress; failure or unmet social expectations, failure to discern thoughts, unhealthy social relationships, unmet or unresolved love, jealousy, loss, and lack of a job ADDIN EN.CITE [33, 38, 39, 42, 48, 50, 51] and psychological distress related to trauma, migration, sexual abuse, trauma, humiliation, and shame [32] • Biological, such as infections (malaria), hereditary diseases, and head injuries ADDIN EN.CITE [20, 37, 39, 53] • Substance use (alcohol and khat); and food poisoning; wondering around where ash or Atela (residual of local beer) had been dumped, or around the tomb; economic problems/poverty ADDIN EN.CITE [32, 33, 38, 39, 41, 50, 53, 54] Quantitative study findings: • of 617 community participants: 44.5% attributed mental illness to head injury; 12.6% to genetic factors; 21.7%) to physical illness; 59.5% to substance misuse; 40.7% to loss of a loved one; 43.9% to conflict with family; 23.7% to punishment by God; 18.1% to evil spirit; and 17.5% to poverty ADDIN EN.CITE [38] • A survey of 435 community members revealed diverse beliefs about the causes of schizophrenia: 67.6% pointed to an anxious personality, 67.1% cited mental illness, 58.9% cited addiction to alcohol or other substances, 54.6% cited both unemployment and failure in love, 53.0% cited work stress, 52.7% cited social issues (loneliness), 26.2% cited heredity/genetics, and from a religious perspective, 54.1% cited God’s will as the cause of schizophrenia [42] • Out of 384 people attending Jimma Hospital, the causes of mental illness were attributed as follows: 51.6% attributed to spiritual possession, 15.9% to evil eye attack, 14.8% to family history, 10.7% to sinful acts, 9.6% to pathogens, 3.4% to stress, and 19.0% were unknown ADDIN EN.CITE [36] • In a sample taken from Ayder Referral Hospital, the perceived causes of mental illness were attributed as follows: stress accounted for 30.5%, possession for 29.3%, walking around garbage dumps for 9%, the evil eye for 9.7%, and sinful acts for 7.3% ADDIN EN.CITE [37]
Pathways to care and help-seeking preference	• Indigenous practices were often chosen as the initial contact sites over modern mental health treatments by both Muslim (witchcraft and herbalists) and Christian (holy water) community members. These practices included prayer, holy water, and consulting with wise men or indigenous healers ADDIN EN.CITE [4, 30, 36–38, 43, 52, 53] • Out of 1044 participants from Amanuel Specialized Mental Hospital, before presenting to the psychiatric hospital, 59% of patients received care from up to four different TFHs. Of those, 30.9% visited a priest, used holy water, or went to church for treatment ADDIN EN.CITE [35] • In a community-based cross-sectional study of 435 participants, almost two-thirds (63.8%) indicated they would seek medical help, the majority (90.8%) would seek religious help, and over half (52.5%) would seek social support for schizophrenia. [42] • From the Mretule Mariam rural community: the intention to seek traditional healers’ help was held by 44.6% of participants. Holy water, prayer, herbal remedies, and wise men were the first-line sources of help ADDIN EN.CITE [33] • Addis Ababa and Assela community members and health care professionals chose modern treatment as their first preference. However, some participants favoured using both or only traditional treatments [55]. Stigma and lack of awareness were reported to be barriers to seeking biomedical care ADDIN EN.CITE [37]
Identification and intervention methods used by TFHs	• All faith healers, diviners, and mixed healers (who used herbs, divining and other forms of spiritual healing practices) reported treating people with mental health conditions ADDIN EN.CITE [13, 18, 20] • Identification of illness was informed by examination of the person’s behaviour, looking for manifestations such as: talking about things that do not make sense, laughing alone, taking off clothes in public places, collecting and carrying dirty things, crying, eating dirty food, and harming or having the intention to harm others. They will also be identified by administering herbal medicine or reading portions of the religious book, *“Kitab-Alherar”* ADDIN EN.CITE [36, 49, 50, 54] • Kallu (the religious leader) was described as investigating the causes of the problem, with no detail about the process, and then providing advice on what to do ADDIN EN.CITE [51] • Spiritual practitioners use holy water, *emnet* (holy ash), *kibakudus* (holy oil), and *mar* (holy honey) in combination with prayer, advice from clergymen, religious books, and the cross, sign of God’s protection against evil spirits. Priests may place the cross on the possessed person or hold it up as he prays and commands the spirits to depart ADDIN EN.CITE [32, 44, 46, 51] • Prayer, herbs/drugs, slaughtering animals as offerings, holy writing, and telepathy following Allah’s or other spiritual guidance, were used by traditional healers ADDIN EN.CITE [52] • Holy water was drunk, a holy book or a piece of metal (usually a knife) was put under a pillow, coffee was brewed, and a priest or sheikh was invited. The sick person may be cauterised with a piece of red-hot iron if the cause is unknown or they may be forced to inhale smoke. A black rooster would be slaughtered if a spirit attack (*likift)* was suspected *(*19*)* [13]
Experience of treatment, satisfaction, gaps and barriers	• People with severe MHCs reported similar satisfaction and perceived benefits from biomedical care and holy water-based healing. However, other faith-based treatments were used less and perceived as being lower in quality with higher reports of harm. For biomedical care, the equivalent lifetime and current access gaps were 41.8 and 59.9%, respectively, while for faith-based and traditional healing (TFH), the gaps were 15.1 and 45.2% [30] • Clients of health centres and clients of TFHs both expressed satisfaction with the consultation, while clients of TFHs expressed higher levels of satisfaction ADDIN EN.CITE [52] • Traditional healers exhibited a mental health literacy, (91.81 ± 10:53.), and age, educational status, family history of a mental illness, and experience of healing people’s mental illness were factors reported. Many healers described mental illness as influenced by supernatural factors, such as possession or spiritual imbalance, which shaped their approach to treatment ADDIN EN.CITE [34]
Collaboration between TFHs and mental health practitioners	• Most people with severe MHCs (92.2%) were comfortable using holy water treatment and medication at the same time, including swallowing their medications with holy water. In terms of ongoing collaborative approaches, 41.3% of patients received one-to-one counselling from priests, 34.3% received psychoeducation and brief eclectic psychotherapy from the mental health service clinic, and 24.4% received counselling support from both [30]. More coordination and collaboration between psychiatrists and religious healers were found after consultative meetings ADDIN EN.CITE [30, 45]. • Except for some resistance from caregivers/family, psychiatric care and holy water healing were reported to be compatible ADDIN EN.CITE [31] • The odds of experiencing a psychotic relapse were 45% lower in people with severe MHCs who sought religious support compared to those who did not seek such support ADDIN EN.CITE [41] • Both modern and traditional treatments were cited as helpful, depending on the mental health condition [55] • TFHs referred people with MHCs for biomedical care and recommended using both biomedical treatments and holy water, after consultative workshops. TFHs comment that the lack of spiritual healing in clinics and biomedical care only controls symptoms, not promoting healing [32]

### Perceived cause of severe mental health conditions (SMHCs)

Twenty studies (six quantitative, twelve qualitative, and two mixed methods) investigated the perceived causes of mental illness from the perspective of people with severe MHCs, community members and TFHs. In most studies, the term “mental illness” referred to severe MHCs as other mental illnesses were not recognised by TFHs. The common attributions for mental illnesses were supernatural forces such as possession by evil spirits, curses, bewitchment (ghosts, malevolent spirits, sorcerers, or magicians), and punishment by God [13, 17, 18, 20, 32–34, 36, 38, 42, 44, 48–53].

Unhealthy social relationships, unrequited love, jealousy, loss, work stress, and lack of work/employment were other important causal attributions [33, 38, 39, 42, 48, 50, 51]. Substance use (alcohol and khat) was also reported to be an important factor in mental ill-health [32, 33, 38, 39, 41, 50, 53, 54]. Worry/fear, work stress, psychological distress related to trauma, migration, sexual abuse, trauma, humiliation, and shame [32, 37, 42, 55], failure, and unmet social expectations, failure to discern thoughts [54], biological causes (such as infections (malaria), hereditary, and head injury) [20, 37, 39, 53], food poisoning [50], wandering around where ash/atela (residue of local beer) had been discarded or around the tomb [20, 37], and economic problems/poverty [20, 32, 33] were also identified as potential causes of mental illness. In a study among seminomadic people of Borana (Oromia), perceived causes of mental health conditions additionally included exposure to wind and thinking too much [52].

Based on quantitative data, Girma and Tesfaye found that among 384 people with mental health conditions (MHC) at Jimma Referral Hospital, 51.6% (198 participants) believed their condition was caused by supernatural possession [36]. In a study of 423 samples taken from Ayder Referral Hospital, the perceived causes of mental illness were attributed to possession (29.3%), evil eye (9.7%), sinful acts (7.3%), walking around garbage dumps (9%), and stress (30.5%) [37]. Both samples for the studies were obtained from tertiary care hospitals, which could introduce bias into the findings.

Two quantitative community-based studies investigated the varied perspectives within the community regarding the perceived causes of schizophrenia. The first was a study by Mekonnen et al. in the rural community of Gondar Zuria district (Maksegnit town), where perceptions of the causes of schizophrenia were investigated among 435 participants. The findings revealed a variety of beliefs about the causes of schizophrenia, with the majority attributing it to an anxious personality (67.6%), mental illness (67.1%), and addiction to alcohol or other substances (58.9%). Participants also commonly cited other perceived causes, such as work stress, unemployment, failure in love, social issues (like loneliness), and God’s will [42]. The second study was conducted in Arbaminch (southern Ethiopia), where 617 participants were surveyed about their perceptions of the causes of schizophrenia. The majority attributed schizophrenia primarily to substance misuse (91.5%) and head injury (68.6%) (Table 3) [38].

Similar to community members, diverse perspectives were held by traditional healers regarding the causes of mental illness. In a quantitative study among 109 traditional healers, the majority (63%) attributed mental health conditions to economic problems and stress due to the loss of belongings [20].

Overall, the findings indicate a range of beliefs among people with MHCs, community members, and TFHs regarding the causes of severe mental health conditions, encompassing supernatural, social and biological causal explanations.

### Pathways to care and help-seeking preferences

Twelve studies (six quantitative, five qualitative, and one mixed methods) investigated help-seeking. In a community-based qualitative study amongst Ethiopian pastoralists, indigenous treatments were preferred for mental health conditions including prayer, holy water (drinking holy water and/or getting baptized with holy water), and consultation with Borana wise men or indigenous healers [53]. Seeking modern mental health care was only used as a last resort. Another study by Alem et al. (1999), based on key informant interviews, reported a similar finding that traditional treatment methods were more commonly preferred for addressing symptoms of MHCs, while biomedical care was for physical diseases and symptoms [4].

According to findings from three community-based studies carried out in rural areas (Arbaminch, Butajira, and Jimma zone) [4, 38, 43]; two studies carried out among people with MHCs from mental health hospitals (Jimma and Mekelle specialised hospitals) [36, 37]; and one study conducted among Entoto holy water users [30], the first-ever contact sites for people with MHCs were faith-based prayer centres, general community traditional healers, holy water, sorcerers/witches, and herbalists.

In a qualitative study conducted among layperson community members, patients from the hospital and their attendants, people from high school, college and others (*n* = 74), healthcare workers, physicians, nurses, psychiatrists and psychiatric nurses (*n* = 35), and traditional healers (*n* = 5) from urban (Addis Ababa) and rural (Assella) areas [55], community members’ first preferences were for biomedical treatment for MHCs, whereas health care professionals preferred either biomedical or combined treatment (biomedical and TFHs) and three out of five traditional healer participants also prefer combined treatment.

Similar results to the qualitative studies were obtained in the other three quantitative studies. Of those attending a tertiary psychiatric hospital in Addis Ababa, more than half (59%) had first sought care from up to four different traditional and faith-based healing providers. Of those, 30.9% went to a church, saw a priest, or used holy water for healing [35]. Another community-based study on the help-seeking behaviours of people with schizophrenia was carried out using case vignettes in Gondar Zone, Maksegnit (district town) [42]. Of the participants, the majority (90.8%) reported that they would prefer religious treatment, more than two-thirds (63.8%) preferred medical care, and more than half (52.5%) responded that they would seek social interventions like getting married, employed, and changing jobs.

In a community-based study carried out in the rural village of Mertule Maryam town using a mixed-method approach and theoretical questions on help-seeking for MHCs, 44.6% of the participants stated that they would first visit conventional healers, such as wise men, herbal treatments, and holy water [33].

### Mental health conditions identification and treatment methods used by TFHs

TFHs reportedly used various techniques to identify people with MHCs. They looked at their behaviours, such as talking about things that did not make sense, laughing alone, taking off clothes in public places, collecting and carrying dirty things, crying, eating dirty food, and harming or having the intention to harm others [49, 54]. Some TFHs gave herbal medicine and recited the “Kitab-Alherar” portion of the holy Quran [50]. The Orthodox Christian faith healers claimed that the clients would identify themselves after hitting them with a holy cross or drinking holy water [13]. Another study from a key informant interview study in Nekemte (Oromia region), reported that Kallu/kallcha (religious leaders) had the skill to investigate the disorder’s causes and advise what to do through ecstatic ritual techniques [51].

Types of treatment differed depending on the type of TFH. Herbalists or *medhanit awaqi*, provided remedies such as herbs, minerals, animal products, and thermal waters. Faith/spiritual healers include diviners (*atmaqi* (baptizer-exorcist), ‘witch doctors’ (*balezar* (zar shaman), *debtera (Orthodox Christians)*, tenquay (sorcerers) or *kalicha (*Muslims) and Ministers in protestant churches exorcised demons. Other practitioners combined herbal remedies and faith-based interventions [13, 18, 20].

Ethiopian Orthodox spiritual/faith practitioners use holy water, *emnet* (holy ash), Kiba Kidus (holy oil), and mar (holy honey) in combination with prayer, religious books, and the cross (utilized as a blessing or to hit persons who are said to be under the influence of spirits) [44, 46, 51]. In a study done in Borana, prayer, herbs or drugs, slaughtering animals as offerings, holy writing, and telepathy were reportedly used as treatment by traditional healers [52]. Other customs were noted by Kortmann F. in 1987, including brewing coffee, inviting a priest or sheik, cauterizing the ill person with a hot iron if the cause was unknown, making the sick breathe smoke, and using a black rooster for those suspects unwell due to *Likift* (spirit attack*)* [19]. At Ghion’s holy water site (now Woliso), Fuller Torrey observed that the public faith healing ceremony began with a reading from the Holy Scriptures, and the faith healer (Abba Wolde Tensae) would then throw handfuls of water in the client’s face and beat the client forcefully with the cross, as a process of identifying and driving out demons, or evil spirits [13]. The author also reported that he observed private consultation services.

### Treatment experience, satisfaction, gaps and barriers

Three studies reported the experience and satisfaction of people with MHCs with different treatment modalities. Shibre T. (2008) compared treatment satisfaction between clients attending traditional healers for physical, emotional, and social problems, and those attending healthcare facilities. Both clients of TFHs and those of health facilities were satisfied with the consultation. However, clients of the traditional healers were more satisfied than the patients in health facilities [52]. Another community-based study of 300 people with psychosis identified through community-based case detection found comparable perceived benefits and satisfaction between biomedical care and holy water but other TFHs, even though they were used less frequently, were thought to be of lesser quality and linked to more harms. This study also reported the treatment gap quantitatively: patients with psychotic illness had 41.8% lifetime and 59.9% current access gaps for biomedical care. However, the gap for traditional and faith-based healing (TFH) was much lower, at only 15.1% for lifetime and 45.2% for the current access gap [40].

One community-based study examined the mental health literacy level of traditional faith healers (TFHs) using a 35-item structured questionnaire called the Mental Health Literacy Scale (MHLS). The study reported that TFHs had a lower mean score on the Mental Health Literacy Scale (MHLS) at 91.81 ± 10.53. The study also reported that factors such as age, educational status, family history of mental illness, and experience in healing people’s mental illness had an impact [34].

### Perspectives and experiences of TFHs and MHPs working together

Seven studies reported a positive attitude towards collaboration between traditional and faith healers (TFHs) and mental health care providers, and a willingness to work together. The process of establishing a collaborative mental health clinic linked to holy water sites at major Ethiopian Orthodox Christian churches (St. Michael’s Church and St. Mary’s Church) was described and evaluated [30]. Most people attending the embedded mental health clinic (92.2%) were comfortable using holy water treatment and medication at the same time, including swallowing their medications with holy water. Regarding ongoing collaborative approaches, 41.3% of the holy water attendants received one-to-one counselling from priests, 34.3% received psychoeducation and brief eclectic psychotherapy from the clinic, and 24.4% received counselling support from both.

Another study from the same programme reported on two-way consultative workshops with faith healers and psychiatrists who participated that aimed to promote transformative learning. The consultative workshops, two-way, resulted in significant changes in their understanding, beliefs, and practices regarding mental health care [45]. They also conducted in-depth interviews to explore TFH’s perspectives and reflections on a collaborative project with biomedical practitioners. TFHs were comfortable referring individuals with MHCs for biomedical care and recommended using both biomedical treatments and holy water for better benefit, after consultative workshops. Some of them expressed concerns about the lack of spiritual healing in clinics and the notion that biomedical care only controls symptoms without curing the illness [32]. These studies underscore the possibility of collaborative methods that involve traditional healers in addressing mental health care gaps in Ethiopia and similar settings. In the study by Asher et al. (2021), interviews were conducted with holy water attendants (who run group houses for holy water residents and are paid by family members) from the same place (Entoto St. Mary holy water) and reported compatible psychiatric care and holy water use except for some resistance from family caregivers [31].

## Discussion

We conducted a scoping review and narrative synthesis to investigate the involvement of traditional and faith healers in the care of individuals with severe MHCs in Ethiopia. We identified 29 studies. The richest evidence pertained to the perceptions of TFHs about the causes of MHCs, some of the healing modalities used and the role of TFHs in the pathway to biomedical care. There was more limited evidence about the detailed processes of care, how care is experienced and the extent to which care from TFHs meets the needs of people with MHCs. Our review identified promising initiatives to develop collaborations between TFHs and mental health services.

Our findings reinforce the observation that traditional and faith healers are widely popular in Ethiopia and have an important part to play in the care of people with MHCs. Health care in Ethiopia is pluralistic, similar to other African countries. It is common for people use a combination traditional, faith-based, and biomedical care for severe MHCs [56, 57]. Though there have been numerous studies conducted on this topic, most studies on traditional faith healers (TFHs) in Ethiopia focused mainly on holy water, while other religious and traditional healers are little studied. Similar to findings from a review conducted in Ghana [58], a substantial number of people visit TFHs as part of their journey towards mental health care. These people prefer to go to TFHs for an initial culturally based assessment (based on their beliefs, customs, and practices) when they require mental health help, advice, or treatment. In our review, there was a report that people visit more than four sites of TFHs before receiving biomedical care [35]. Nonetheless, when mental health care was made locally available, it appears that people were comfortable using either TFHs or biomedical care or using both, including swallowing the medication with holy water [30, 31]. There was also a report of reduced relapse in people with severe MHCs who used both [41]. Most studies did not allow for an in-depth understanding of the roles of TFHs. Even those studies that used focused ethnography were brief in nature and provided little information about the processes of healing practices and the perspectives of people with mental health conditions.

TFH services were not accessible to all and were not acceptable to all, and there were variable views and experiences including some harmful and odd practices like cauterizing and beating. In a previous randomized controlled trial (RCT) conducted among individuals with mental health conditions at a large prayer camp in Ghana, it was observed that the intervention group, which received both medications and participated in prayer camp activities, showed a reduction in symptoms compared to the group that received only the usual treatment, which included prayer camp activities, at six weeks. However, there was no reduction in the number of days spent in chains, which is a matter of significant clinical and human rights concern [59]. The authors noted incomplete integration of the medical team into decision-making by the prayer camp staff, who made all final decisions about chaining.

The variation in acceptance of TFHs can be based on the type of healer involved. It is difficult to determine if individuals choose traditional faith healers (TFHs) out of genuine preference or due to a lack of other options. Studies conducted by Hailemariam et al., particularly within the context of the Programme for Improving Mental Health Care (PRIME), illustrate that when biomedical care is accessible, there is a high level of engagement with it. Their findings suggest that the presence of TFHs does not necessarily result in higher dropout rates from biomedical care. Instead, when psychiatric services are available and easily accessible, people often make extensive use of them [60].

The experience of individuals with mental health conditions (MHCs) seeking care from traditional faith healers (TFHs) is not well understood. Research by Souraya et al. has revealed that those with severe MHCs in Ethiopia often have limited decision-making power regarding their treatment. Instead, it is their families who make decisions about seeking help from TFHs. This knowledge gap hinders our understanding of the experience from the perspective of the person with the MHC [61]. In Ethiopia, there is still a significant treatment gap for those with severe MHCs [40]. The access is not limited to biomedical care but the community does not always have equal access to TFHs everywhere [40]. Researchers, including the World Health Organization (WHO) and the Ethiopian Federal Ministry of Health (FMoH), recommended establishing collaboration between Traditional and Faith Healers (TFHs) and biomedical health services [21, 62], to narrow the treatment gap, gain community acceptance, and reduce harmful practices [1, 63, 64]. Studies about collaboration between MHPs and TFHs in the present review confirm the viability of such collaboration and the complementarity of TFHs (holy water healers) and biomedical sectors of mental health care as a method of enhancing access to services [30, 45]. In these studies, misconceptions or “disorienting dilemmas” from both sides of the providers were resolved through a series of consultative workshops that were guided by the transformative learning theory (through dialogue and critical reflection, changing preexisting perceptions, cognitive processes, and behaviour). However, those studies were only specific to the Ethiopian Orthodox Christian holy water sites. The process for collaboration is not specified; only its benefits, opportunities, and challenges are outlined. Because of our limited understanding of the processes of healing and care, and experiences of care, our understanding of the most productive and ethical way to work together remains limited.

## Limitations

Despite efforts to complete a comprehensive search, relevant studies may have been missed due to variations in terminology or indexing, as we do not have a common definition of severe mental health conditions.

### Implications

Our review has highlighted important gaps in the literature on the role of TFHs in healing and care for people with severe MHCs in Ethiopia. Existing studies indicate the potential for TFHs and biomedical mental health care providers to collaborate, to reduce delays and increase coverage for biomedical care but also to more holistically respond to the needs of people with MHCs. Further research is required to foreground the voices of people with severe MHCs and their experiences of different healing modalities, to understand the pluralistic care processes and the challenges and opportunities of collaboration between TFHs and MHPs in Ethiopia.

## Conclusion

Although much is known about the place of TFHs within care pathways for people with mental health conditions in Ethiopia, there are evidence gaps in relation to the perspectives of people with mental health conditions and a rich contextual understanding of processes and mechanisms of healing that need to underpin plans for collaboration.

## Supplementary Information

Supplementary Material 1

## Data Availability

The data supporting the findings of this study are available as a supplementary file accompanying the manuscript.

## Abbreviations

ASSIST: Alcohol smoking and substance involvement screening test
BMPs: Bio-medical practitioners
CMQS: Causal models questionnaire for schizophrenia
BPRS: Brief psychiatric rating scale
FGD: Focus group discussion
GHQ: General help seeking questionnaire
JBI: Joanna briggs institute
MARS: Medication adherence rating scale
MHCs: Mental health conditions
MHP: Mental health providers
mhGAP: mental health GAP
mhGAP-IG: mental health Gap-Intervention guide
OSF: Open science framework
PCC: Population concept context
PRISMA-ScR: Preferred reporting items for systematic reviews and meta-analyses scoping review extension
QuADS: Quality assessment for diverse studies
SMDs: Severe mental disorders
TFHs: Traditional and faith healers
WHO: World health organization
