# Supplementary material for: Experiences and perspectives on traditional and faith healers’ involvement in the care of people with severe mental health conditions in ethiopia: a scoping review
Source: Int J Ment Health Syst. 2025 Dec 8;20:1. doi: 10.1186/s13033-025-00691-9 (PMC12797701; doi:10.1186/s13033-025-00691-9)
Supplement: Supplementary file 1 — Supplementary Material 1 [file 13033_2025_691_MOESM1_ESM.docx]

The list of Tables is to be added as a supplementary file.

Table 1: data extracted from 31 studies.

| Author/year | Location | Methodological approach | Participants | Research questions/aims | Main theme/finding |
| --- | --- | --- | --- | --- | --- |
| **Qualitative studies** | | | | | |
| Alem A. et al. 1999 | Butajira | 100 Key Informants (KI) interviews.  -WHO KI questionnaire, and case vignettes of seven common neuropsychiatric disorders (Epilepsy, Schizophrenia, Acute psychosis, Mental retardation, Neurosis, Mania, and Severe depression) were used. | Community leaders and religious leaders selected from Nine peasant associations (PAs) and one urban dweller’s association (UDA) of Butajira, rural people of Ethiopia | Mental health help-seeking and preferred interventions | - Traditional treatment methods were preferred more often for treating symptoms of mental disorders and modern medicine was preferred more often for treating physical diseases or symptoms.  - Muslim informants prefer witches and herbalists, while Christian informants prefer holy water more. |
| Anderson L. 2007 (preprint) | Lalibela | -literature review (articles and religious books), interview of priests and community members of Lalibela, and observation of holy water users at church.  - all fieldwork completed in one week. | Lalibela community members, priests, and the patients cured with Church healing | To examine the healing practice of the Ethiopian orthodox church and its relationship with other THs | -P**erceived cause of mental illness**: supernatural forces (ghosts, malevolent spirits, sorcerers, or magicians).  -**Support of biblical literature**: The Holy Bible is the base and source of all kinds of good knowledge that is prevailing in the world, including miraculous cures. (In old and new testaments)  - **spiritual treatment practices** such as holy water, Emnet (ash), Kiba kudus (holy oil), and Holy honey in combination with prayer and the cross. |
| Asher, L. et al 2021 | Addis Ababa | A qualitative study of the holy water attendants’  role and attitudes as the main study using:  -Clinic records, Semi-Structured questionnaires, and interview guide questions were used.  -Purposive sampling for attendants and snowballing for service users were used. | Holy water attendants and service users of Entoto holy water healing | The practice of holy water healing for mental illness | -Psychiatric care was felt to be compatible with holy water by some, but not all, attendants.  -Family members often held the "final say" in whether individuals attended the clinic, and in some cases, they strongly opposed the use of psychiatric care.  -from the clinic record the most common diagnosis (73.3%) was schizophrenia.  - holy water attendants are best considered as gatekeepers. |
| Bahretibeb Y et al. 2024 | Addis Ababa | In-depth interview | Holy water healers of St Michael’s and St Mary’s churches in  Entoto | To examine religious healers' explanatory models of mental illness, general treatment approaches, and their perception, attitude, and willingness regarding cross-system collaboration. | **-Perceived causes:** the most prevalent were in references to the Bible, demonic possession, evil spirit witchcraft and bewitchment. Other causes include being cursed by the elderly, excessive alcohol use, khat use, drug abuse, heredity, physical illnesses, injury and old age.  - socioeconomic problems and psychological distress related to trauma, migration, sexual abuse, trauma, humiliation, shame, and poverty.  **-Collaboration**: multiple treatments were preferred holy water, prayers and advice from clergymen, and Western treatment to attain maximum benefits.  **-Experience collaboration**: Referrals, the people with MHCs were sent for biomedical care and healers recommended using both biomedical treatments and holy water, which proved to be more effective.  - Overall, they have a positive view of collaboration. Pointing out that the lack of spiritual healing in clinics and biomedical care only controls symptoms, not promotes healing. |
| Baheretibeb, Y. et al 2022 | Addis Ababa | Focused ethnography:  Including examination of recordings and minutes of stakeholder meetings, workshops, and informal  interviews with participants, and finally analysed for evidence of Mezirow’s 11 stages of  of transformative learning. | Psychiatrists. psychiatric trainees, and healers from two holy water sites | Collaboration between faith  healers and biomedical practitioners | After 11 phases of Transformative Learning Theory (TLT); mental health care delivery with coordination and collaboration between psychiatrists and faith healers is possible and has the potential to reduce stigma and improve access to care. |
| Baye Berihun 2015 | Addis Ababa | -In-depth interviews with four religious healers and two clients and observation of the treatment process | Entoto Kidane-Mihret Monastery priests and patients | Faith as a means of healing | -**Perceived cause of mental illness**: it is considered a trick in the manifestation of the demons.  -The treatments include Holy Water, Holy Oil, Cross, and Holy Ash.  -treatments are to flush or drive out the possessing demons from their host. |
| Desalegn A. et al 2016(preprint) | Gondar | -Data collected by using Interview guide questions and observation  -8 traditional healers and 2 clients were selected purposively. | Traditional healers (THs) and their clients with mental illness | Conceptualisation of mental illness and treatment practice among traditional  healers | -Perceived causes of mental illness: God’s will, Evil eye, Bewitchments, Demonic possessions, stress, economic problems, family conflicts, death of relatives, and substance use.  - **Identification of the mentally ill**: using religious books, herbal plants, observation of client behaviour  -**Treatment practised:** herbal medicines, spiritual treatments (holy water, holy bibles) traditional rituals, and consultations  -Traditional healers (THs) are volunteers who work with biomedical care providers. |
| E. Fuller Torrey  1972 (book chapter) | Waliso (Oromia) | Ghion holy water healer (Abba Wolde Tensaye) is found in the Oromia region near Addis Ababa 130 kilometres away.  -he did observation and informal interviews with the healer and community around. | Ghion holy water healer (Abba Wolde Tensaye) | The practice of religious healers at the holy water site | **Perceived cause:** Supernatural, mostly spirit attacks when the individual is alone around a river, forest, or cave.  Treatment practice: public and private using holy water and holy cross.  Private: suggestion, confession, aversive conditioning, and hypnosis-like environmental manipulations. |
| Gutema, B. T. and  Mengstie, M. M.  2022 | Berta community in Assosa, | -7 participants were selected through snowball sampling and interviewed using semi-structured questionnaires (about how they identify MI, perceived cause, and treatment they used for MI) | Traditional healers | Traditional  healers’ perception of the causes of mental illness,  ways and signs of identifying mentally ill persons and  treatment practices | **-perceived causes of mental illness**; curses/witchcraft, unhealthy social relationships, jealousy, hereditary, substance use, punishment of God, and food poisoning.  -**Identification of the mentally ill**,  1. Looking at his /her behaviour like talking about things that don’t make sense laughing alone, taking off clothes in public places, collecting and carrying dirty things, crying, eating dirty food, harming or intending to harm others, being aggressive…  2. by giving herbal medicine and using religious books (Kitab-Alherar)  -THs uses herbals, religious books, and bone divination to guide their treatment of persons with mental illnesses. |
| Jacobsson, L.  Merdasa, F. 1991 | Nakamte, Oromia | Before actual interviews (1969-1971) information was collected from patients, healthcare workers in hospitals and health centres in the area, and colleagues.  Three months were spent in the field. | Coptic priests, Muslim sheikhs, and other traditional healers | To describe perceptions  of mental disorders and the different  kinds of traditional healers and treatments | -In the traditional Oromo society, Kallu (religious leader) investigates the causes of mental disorders and advises what to do.  -perceived cause of mental illness: disturbances in the relationship, divinity, possession by evil spirits, and God to punish unfaithfulness.  -Treatments: priests and monks praying and giving holy water or eventually exhortation.  - Some Muslim sheikhs treat people with an apparent mental disturbance with prayers and herbal remedies. |
| Kortmann F. 1987 | Ethiopia | Participants were asked to write an essay based on Guiding questions.  Clinical experience and literature consultation were supplemented. | 21 medical students who had just completed Psychiatry training. | Overviews of traditional and professional mental health care in Ethiopia | -Perceived cause of mental illness: attack from Saytan (Devil), evil eye, Digmt by a person who has contact with the devil; like a magician and Debtera(Islamic equivalent Kabir); Adbar(type of spirit), and Zar(inherited spirits).  -Treatment practice: holy water, a holy book, a piece of metal (usually a knife) is put under a pillow, Coffee is brewed, a priest or sheikh is invited, cauterize the sick person is with a piece of red-hot iron the cause is unknown, forcing sick to inhale smoke, black rooster for sick suspected by *likift*, |
| Monteiro NM, Balogun SK 2013 | Addis Ababa and Asella | Qualitative information was gathered through interviews using interview guide questions (IGQs) from 115 participants: laypersons(N=75), healthcare workers(N=35), and traditional healers(N=5). | Community members, healthcare workers, and traditional healers of urban (Addis Ababa) and rural (Assella) areas. | Traditional perceptions and treatment of mental disorders in Ethiopia | -perceived Cause of mental illness: excessive worry/fear, excessive thinking,  -Both modern and traditional treatments were cited as helpful, depending on the disorder.  -Regarding first preference 46% said that modern treatment was best, 16% preferred traditional treatment and 33% said both |
| Robert Giel 1968 | Showa province | Ethnography: used Priest’s recording and Observation | Holy water healers, and service users | Faith-healing and spirit-possession in Ghion, Ethiopia | -People visit Ghion for organic illnesses, mental illness, and in case of social misfortune.  -The cure is complex, often gradual, and a combination of preceding medical treatment and the services offered by the priest. |
| Seblewengel D. et al 2016 (thesis) | Addis Ababa | -Participants selected purposively (4  vocational ministers, 4 gifted healers; 2  each denomination)  -primary, secondary, and web-based literature were consulted, and semi-structured interviews and **observation** (for 13 hours) were made. | Ministers, healers, and service users of Kale Heywet Church and Muluwongel Believers’ Church | Perceived causes, diagnosis methods, and treatment practices of mental illness of Evangelicals | -perceived causes of mental illness: sin, satanic attacks, failure & unmet social expectations, failing to discern thoughts, substance abuse,  -treatment: counselling, prayer, referral  **-Identification of the mentally ill**: observation, prayer, reading of the Bible, and the gift of discernment.  -Ministers did not deny the importance of biomedical care. |
| Shibre T. et al 2008 | Butajira | Through Mapping: using key informant interviews 24 THs were identified. Then using semi-structured questionnaires THs, clients of THs, and patients of health centers (HCs) were interviewed. | Traditional Healers (THs), service users of THs, and patients of health centres | traditional treatment of mental illness; perception, practice, and satisfaction | -THs reported cause of emotional problems is supernatural forces (Allah, Satan, and evil spirits)  -All healers believed they were able to treat any emotional and physical illness.  -THs sites like prayer, herbs/drugs, slaughtering animals as offerings, holy writing, and telepathy were used by many people.  -Both clients of THs and patients of health centres were satisfied with the consultation, but the clients of the THs were more satisfied than the patients in health centres. |
| Tadesse Z. et al 2018  (Thesis) | Addis Ababa | -Informal and in-depth interviews, **observation** at service sites, and review of secondary data were used for data collection.  -Participants were selected Purposively. | -Participants were Patients at Shinkuru Mikael church but family members, friends, neighbours, and religious personnel were also used for informal interviews. | To explore the process of holy water therapy for mental disorders | -The Holy Water has attendants(association), for “difficult cases” to provide care (Receive about 1000 birr based on the ability of the family to pay).  -**cause of mental illness**: psychological, social, and supernatural phenomena.  -Healers can identify the mental illness of those possessed by demons.  -Patients are ordered to drink holy water and pray every day.  -Patients who come with a chain will be unchained after they show some improvements.  -The healing process depends on the nature of the ailments. |
| Tefera S. & shibrie T. 2012 | Borena | Purposively selected key informants from three villages and conducted six FGDs (separate for three for males and three for females) | Borena seminomadic people | Perceived causes of severe mental disturbance and preferred interventions | -**supernatural causes**: possession by evil spirits, curses, bewitchment, ‘exposure to wind’ and  -biopsychosocial cause: infections (malaria), loss, ‘thinking too much’, and alcohol and khat abuse.  -The favoured treatments: Prayer, holy water treatment, consultation with Borana wise men or indigenous healers, and, as a last resort, seeking modern mental health care. |
| **Mixed method studies** | | | | | |
| Anbesaw T. et al 2024 | Dessie | Qualitative, in-depth interviews and focused group discussion using interview guide questions and quantitative using the Mental Health Literacy Scale (MHLS) is a 35-item questionnaire. Social Support Scale (The Oslo 3-items) was also used | All traditional healers in Dessie town | Traditional Healer’s Mental Health Literacy and Factors Associated | **-Perceived cause**: possession by evil spirits, curses, bewitchment, excessive substance use  -The mean total mental health literacy score was found to be 91.81 ± 10:53.  -factor affecting MHLS include age, educational status, family history a mental illness, and experience of healing people's mental illness |
|  |  |  |  |  |  |
| Yeshanew B. Et al. 2020 | East Gojjam | Mixed method: 964 participants were selected by multi-stage sampling technique.  -Structured interviews using a General Help-Seeking Questionnaire  (GHSQ) and focused group discussions were used for data collection. | Community participants of Mertulemaryam town | Help-seeking for mental health problems | -**cause of mental illness**: evil spirit, violation of God’s doctrines and testaments, loss of loved one, poverty, too much thinking, and substance use  - 81.5%) were likely to seek help from healthcare workers and 44.6% of participants had the intention to seek help from traditional healers.  -help-seeking sites: holy water, praying, and herbal treatments, wise men.  -factors for help-seeking age, marital status,  social support, source of information, perceived severity of mental illness and perception |
| **Quantitative studies** | | | | | |
| Baheretibeb Y. et al 2021 | Addis Ababa | Quantitative report: workshops and Examination of basic clinical records of 1888 people with MHCs over 7 years at the newly created clinic. | Attendees of St. Michael’s Church and St Mary’s church and psychiatrists and psychiatric trainees | Active collaboration between biomedical and TFHs, usage of the newly created clinic. | -88% of people with MHCs first sought help at TFH. Among them, 48% also had sought biomedical care, with 10.8% had been still receiving follow-up. others discontinued due to side effects (55%), unaffordable costs (23%), refusal to take medication (10%), and lack of improvement (7%).  -However, many of these individuals were eventually referred to the clinic, 26% were directly referred by priests, 13% by holy water treatment attendants, and 7% sought help at the clinic on their own. The other 54% of the clinic’s patients were brought to the clinic by family members.  -92.2% of patients were comfortable using holy water treatment and medication at the same time, including swallowing their medications with holy water.  - 41.3% of patients received one-to-one counselling from priests, 34.3% received psychoeducation and brief eclectic psychotherapy from the clinic, and 24.4% received counselling support from both. |
| Bekele Y. et al 2009 | Addis Ababa | -1044 participants, new patients (never history of visits to the hospital) from May to June 2023 were included consecutively.  -The interviews were conducted by trained psychiatric nurses.  - using structured WHO encounter form questionnaires + sociodemographic variables. | Patients with a new presentation to Amanuel Specialized Hospital | Pathway to psychiatric care | -41% come directly to psychiatric care,  -59% of patients sought care from up to four different caregivers before arriving at the psychiatric hospital.  -Of these, 30.9% of patients seek care from priests/holy water/church. |
| Belachew Y. et al 2019 (preprint) | Sodo District | descriptive quantitative study:  -participants were identified through household census where names and the predominant type of healing were listed by HEWs.  -face-to-face interviews of 173 traditional healers using structured questionnaires prepared about healing practices, clients’ characteristics, experience, and intention of collaborations. | Traditional healers from Sodo | Pathways to Psychiatric Care in Ethiopia | - Four types of healers were identified- herbalists, faith healers mixed herbal and faith practitioners, and diviners. -Half the healers entered the healing practice due to family kinship, whereas 26(15%) were due to ancestral spirit.  -One hundred nine (63%) of the healers reported that their clients visit both TFHs and biomedical practices. All faith, diviners, and mixed healers treat mental illness, but herbalists don’t.  -Each healer sees 4-20 clients per day. all faith healers have a residence for their clients. |
| Boti, N. et al. 2020 | Arbaminch | Quantitative study: 617 Samples were selected randomly,  -The authors developed structured interviewer-administered questionnaires and case vignettes were used. | community survey | community perception and attitude towards  people with schizophrenia | -Perceived cause: 275 (68.58%) of participants head injury, 78 (19.45%) genetic, 135 (33.67%) physical illness, 367 (91.52%) substance misuse, 251 (62.59%) loss of loved one, 271 (67.58%) conflict with family, 146 (36.50%) punishment by God, 112 (27.90%) evil spirit, and 108 (26.93%) poverty.  -More than 70% of the sample have good perceptions and attitudes towards schizophrenia.  -participants preferred spiritual or traditional methods for the treatment of schizophrenia. |
| Fekadu W. et al. 2015 | Addis Ababa | 416 participants were selected randomly and interviewed using a structured questionnaire (WHO’s ASSIST) and using the Brief Psychiatric Rating Scale (BPRS). | Entoto St. Mary holy water users | prevalence and associated factors of mental illness among  holy water users | -based on BPRS 60.1% of participants have some mental illness.  -of those 18.5% had a known  history of previous psychiatric illness.  - 7.79% of those mentally ill are taking medication in the holy water.  -Joblessness, substance use, and having medical illness were factors prone to mental illness. |
| Fekadu A. et al 2019 | Butajira | 300 psychotic patients were identified through community-based case detection and confirmed through subsequent structured clinical evaluations.  Interview with Butajira Treatment Gap Questionnaire (TGQ) | Identified psychotic patients | Psychosis treatment gap and its consequences | -Lifetime access gap: 41.8% for biomedical care, 15.1% for TFHs.  -current access gaps: 59.9% for biomedical care, 45.2% for TFHs.  -The perceived benefit and satisfaction measuring the quality of care from biomedical care and a specific type of FTH (holy water) were comparable. However biomedical care was preferred compared to other types of FTH. |
| Fikreyesus, M. et al 2016 | Jimma | 386 Participants were invited for an interview consecutively.  -Face-to-face interviews were conducted using sociodemographic variables and the Medication Adherence Rating Scale (MARS). | Psychotic patients of Jimma  University specialized hospital | prevalence of relapse and associated factors among patients with psychotic disorders. | -When patients reported seeking religious assistance, their chances of experiencing a psychotic relapse were 45% lower than those of patients who did not.  -Patients who had high social support score was 48 % lower than that of patients who had low social support score |
| Mekonen E. G. et al 2020 | Gondar | systematic sampling techniques to select households and simple random sampling to select 435 eligible participants.  -Face-to-face interview using socio-demographic variables and Causal Models Questionnaire for Schizophrenia (CMQS) | 435 Community- Gondar Zuria district, Maksegnit town | Perceived Cause and determinants of help-seeking Behaviour of schizophrenia | -Perceived cause of schizophrenia: 67.1% believe due to mental illness, 26.2% heredity/genetics, 53.0% work stress, 54.6% unemployment, 54.6% failure in love, 52.7% social issue (loneliness), 67.6% anxious personality, 58.9% alcohol or other addiction, and from the religious perspective, 54.1% God's will.  -help-seeking: 63.8% reported that they will take to health institutions, 90.8% religious healing (praying and holy water), and 52.5% social help (getting married, employed or changing jobs)  -perceived religious cause for schizophrenia increases religious help-seeking. |
| Girma, E. and Tesfaye M. 2011 | Jimma | 384 Participants with new episodes of mental illness were selected consecutively.  -Data was collected through Chart reviews and face-to-face interviews using structured questionnaires. | Patients from Jimma University Hospital | Patterns of treatment-seeking behavior for  mental illnesses | - Perceived Causes of Mental Illness: 198 (51.6%) of participants perceive due to spiritual possession, 61 (15.9%) due to evil eyes attack, 57 (14.8%) having a family history, 41 (10.7%) having sinful acts, 37 (9.6%) due to pathogens, 13 (3.4%) due to stress, and 73 (19.0%) do not know.  -The initial source of help for mental disorders was traditional healers.  -98.7% of patients believe their illness will be cured with medication. |
| Selamu M. et al 2015 | Butajira | Quantitative data were collected using a community resource inventory by trained Health Extension Workers (HEWs). | Community survey | To map community resources available for mental health care | -Traditional healers (49 herbalists, and 21 Tenqway/witch doctors’), 27 holy water sites, and other community-based organizations were identified. |
| Tesfaye, Y.  Et al 2020 | Jimma | Community-based survey  -423 samples were selected using systematic random sampling.  -Data collected by face-to-face interviews using structured questionnaires (sociodemographic and General Help-seeking  Behavior questionnaire (GHSQ)) | Jimma Zone, Seka Chekorsa district | Patterns of treatment-seeking behavior for mental illnesses | -The overall help-seeking behaviour score: only 38.8% of the respondents had good help-seeking behaviour towards mental health problems.  -respondents preferred non-medical treatment approaches more (48.4% traditional and 64.8% religious healers). |
| Teshager, S. et al. 2020 | Mekele | -423 patients with mental illnesses were selected consecutively.  -Sociodemographic variables and the WHO Encounter Form were used for data collection. | Patients attending Ayder Hospital | Pathways to psychiatric care and factors  associated with delayed help-seeking | **Perceived causes of mental illness**: spiritual possession, evil eye, sinful act, walking around garbage dumps and stress accounted for 124 (29.3%), 41 (9.7%), 31 (7.3%), 38 (9%) and 129 (30.5%), respectively.  -**Referral pathway**: family, relatives (59.6%), former patients (28.1%), religious healers (71.4%), psychiatry clinics (22.5%), and traditional healers (2.6%) were the first source of help for mental illness.  **Reasons for delay in help-seeking:** Distance, Financial difficulties, Lack of mental health service, did not believe in modern treatment, |

Table 2: Quality assessment of included studies (quality appraisal) using Quality Assessment for Diverse Studies (QuADS)

| Articles | Quality Assessment for Diverse Studies (QuADS) Items | | | | | | | | | | | | | | Score/ 39 |
| --- | --- | --- | --- | --- | --- | --- | --- | --- | --- | --- | --- | --- | --- | --- | --- |
|  | 1 | 2 | 3 | 4 | 5 | 6 | 7 | 8 | 9 | 10 | 11 | 12 | 13 |  | |
| Qualitative studies | | | | | | | | | | | | | | | |
| Alem A. et al. 1999 | 3 | 3 | 3 | 3 | 3 | 3 | 3 | 3 | 3 | 1 | 2 | 0 | 0 | 30 | |
| Anderson L. 2007 | 2 | 3 | 2 | 3 | 1 | 2 | 1 | 2 | 2 | 1 | 2 | 0 | 3 | 24 | |
| Asher, L. et al 2021 | 3 | 3 | 3 | 3 | 2 | 3 | 3 | 2 | 3 | 3 | 3 | 0 | 3 | 35 | |
| Baheretibeb, Y. et al 2024 | 3 | 3 | 3 | 3 | 2 | 3 | 3 | 3 | 2 | 3 | 3 | 0 | 3 | 30 | |
| Baheretibeb, Y. et al 2022 | 3 | 3 | 3 | 3 | 2 | 2 | 2 | 3 | 2 | 3 | 3 | 0 | 3 | 32 | |
| Baye Berihun 2015 | 3 | 3 | 3 | 1 | 1 | 3 | 2 | 3 | 2 | 2 | 2 | 0 | 3 | 28 | |
| Desalegn A. et al 2016 | 3 | 3 | 2 | 2 | 1 | 3 | 2 | 3 | 2 | 2 | 2 | 0 | 2 | 27 | |
| Gutema, B. T. and Mengstie, M. M. 2022 | 3 | 3 | 3 | 2 | 2 | 2 | 2 | 3 | 3 | 3 | 3 | 0 | 0 | 29 | |
| Jacobsson, L. & Merdasa, F. 1991 | 3 | 3 | 3 | 2 | 1 | 1 | 2 | 2 | 3 | 1 | 2 | 0 | 0 | 23 | |
| Kortmann F. 1987 | 3 | 2 | 2 | 2 | 1 | 2 | 2 | 2 | 2 | 2 | 3 | 0 | 2 | 25 | |
| Monteiro NM, Balogun SK 2013 | 3 | 3 | 3 | 2 | 1 | 1 | 2 | 3 | 3 | 3 | 3 | 0 | 2 | 29 | |
| Robert Giel 1968 | 2 | 3 | 2 | 3 | 1 | 2 | 2 | 1 | 1 | 2 | 2 | 0 | 0 | 21 | |
| Seblewengel D. et al 2016 | 3 | 3 | 3 | 3 | 2 | 2 | 2 | 2 | 2 | 2 | 2 | 0 | 1 | 27 | |
| Shibre T. et al. 2008 | 3 | 3 | 3 | 3 | 3 | 3 | 3 | 3 | 3 | 3 | 3 | 0 | 3 | 36 | |
| Tadesse Z. et al 2018 | 3 | 3 | 3 | 3 | 3 | 2 | 2 | 2 | 2 | 2 | 2 | 0 | 2 | 29 | |
| Tefera S. & shibrie T. 2012 | 3 | 3 | 3 | 3 | 3 | 3 | 3 | 3 | 3 | 3 | 3 | 0 | 2 | 35 | |
| Mixed method studies | | | | | | | | | | | | | | | |
| Anbesaw T. et al 2024 | 2 | 3 | 2 | 2 | 3 | 2 | 1 | 2 | 2 | 2 | 3 | 0 | 2 | 22 | |
| Yeshanew B. Et al. 2020 | 2 | 3 | 2 | 2 | 2 | 2 | 2 | 2 | 3 | 2 | 2 | 0 | 2 | 26 | |
| Quantitative studies | | | | | | | | | | | | | | | |
| Baheretibeb Y. et al 2021 | 3 | 3 | 3 | 3 | 3 | 3 | 3 | 3 | 3 | 3 | 3 | 0 | 3 | 36 | |
| Bekele Y. et al 2009 | 3 | 3 | 3 | 2 | 2 | 2 | 3 | 2 | 3 | 3 | 3 | 0 | 3 | 32 | |
| Belachew Y. et al 2019 | 3 | 3 | 3 | 1 | 3 | 2 | 2 | 3 | 3 | 2 | 2 | 0 | 0 | 27 | |
| Boti, N. et al. 2020 | 2 | 3 | 3 | 2 | 2 | 2 | 2 | 3 | 3 | 3 | 3 | 0 | 3 | 31 | |
| Fekadu W. et al. 2015 | 2 | 3 | 2 | 2 | 3 | 2 | 2 | 3 | 2 | 3 | 3 | 0 | 2 | 29 | |
| Fekadu A. et al 2019 | 3 | 3 | 3 | 3 | 3 | 3 | 3 | 3 | 3 | 3 | 3 | 1 | 3 | 37 | |
| Fikreyesus, M. et al 2016 | 2 | 2 | 1 | 2 | 1 | 1 | 2 | 1 | 2 | 2 | 1 | 0 | 2 | 19 | |
| Mekonen E. G. et al 2020 | 2 | 2 | 2 | 2 | 3 | 2 | 2 | 2 | 2 | 2 | 2 | 0 | 2 | 24 | |
| Girma, E. and Tesfaye M. 2011 | 3 | 2 | 2 | 2 | 2 | 2 | 2 | 2 | 2 | 3 | 2 | 0 | 2 | 26 | |
| Selamu M. et al 2015 | 3 | 3 | 3 | 3 | 3 | 2 | 3 | 3 | 3 | 3 | 3 | 1 | 3 | 36 | |
| Tesfaye, Y. et al 2020 | 2 | 2 | 2 | 2 | 2 | 2 | 2 | 2 | 1 | 2 | 2 | 0 | 0 | 21 | |
| Teshager, S. et al. 2020 | 2 | 3 | 3 | 3 | 3 | 2 | 2 | 2 | 2 | 2 | 2 | 0 | 3 | 31 | |

**NB**: 0 = Not at all 1 = Very slightly 2 = Moderately 3 = Complete,

Item 1. Theoretical or conceptual underpinning to the research. 2. Statement of research aim(s). 3. Clear description of research setting and target population. 4. The study design is appropriate to address the stated research aim(s). 5. Appropriate sampling to address the research aim(s). 6. Rationale for choice of data collection tool(s). 7. The format and content of the data collection tool are appropriate to address the stated research aim(s). 8. Description of the data collection procedure. 9. Recruitment data provided. 10. Justification of analytic method selected. 11. The method of analysis was appropriate to answer the research aim(s). 12. Evidence that the research stakeholders have been considered in the research design or conduct. 13. Strengths and limitations critically discussed.

Table 3: Pubmed Search conducted on May 14, 2024

| Search | Query | Result |
| --- | --- | --- |
| #4 | ("tradition"[All Fields] OR "tradition s"[All Fields] OR "traditional"[All Fields] OR "traditionals"[All Fields] OR "traditions"[All Fields] OR ("spiritual"[All Fields] OR "spiritualism"[MeSH Terms] OR "spiritualism"[All Fields] OR "spirituality"[MeSH Terms] OR "spirituality"[All Fields] OR "spiritualities"[All Fields] OR "spirituality s"[All Fields] OR "spiritually"[All Fields] OR "spirituals"[All Fields]) OR ("folk"[Journal] OR "folk"[All Fields]) OR ("indigene"[All Fields] OR "indigeneity"[All Fields] OR "indigeneous"[All Fields] OR "indigenes"[All Fields] OR "indigenization"[All Fields] OR "indigenous"[All Fields]) OR ("religious"[All Fields] OR "religiously"[All Fields] OR "religiousness"[All Fields]) OR ("contemporaries"[All Fields] OR "contemporary"[All Fields]) OR ("alternance"[All Fields] OR "alternances"[All Fields] OR "alternant"[All Fields] OR "alternants"[All Fields] OR "alternate"[All Fields] OR "alternated"[All Fields] OR "alternately"[All Fields] OR "alternates"[All Fields] OR "alternating"[All Fields] OR "alternation"[All Fields] OR "alternations"[All Fields] OR "alternative"[All Fields] OR "alternatively"[All Fields] OR "alternatives"[All Fields]) OR ("holistic"[All Fields] OR "holistically"[All Fields]) OR "multifaith"[All Fields]) AND ("heal*"[All Fields] OR ("medicin"[All Fields] OR "medicinal"[All Fields] OR "medicinally"[All Fields] OR "medicinals"[All Fields] OR "medicine"[MeSH Terms] OR "medicine"[All Fields] OR "medicine s"[All Fields] OR "medicines"[All Fields]) OR "remed*"[All Fields] OR "therap*"[All Fields] OR ("assistances"[All Fields] OR "assistant s"[All Fields] OR "assistants"[All Fields] OR "assisted"[All Fields] OR "assisting"[All Fields] OR "assistive"[All Fields] OR "dental assistants"[MeSH Terms] OR ("dental"[All Fields] AND "assistants"[All Fields]) OR "dental assistants"[All Fields] OR "assistant"[All Fields] OR "helping behavior"[MeSH Terms] OR ("helping"[All Fields] AND "behavior"[All Fields]) OR "helping behavior"[All Fields] OR "assist"[All Fields] OR "assistance"[All Fields] OR "assists"[All Fields]) OR "intervention*"[All Fields] OR ("therapeutics"[MeSH Terms] OR "therapeutics"[All Fields] OR "treatments"[All Fields] OR "therapy"[MeSH Subheading] OR "therapy"[All Fields] OR "treatment"[All Fields] OR "treatment s"[All Fields]) OR ("assess"[All Fields] OR "assessed"[All Fields] OR "assessement"[All Fields] OR "assesses"[All Fields] OR "assessing"[All Fields] OR "assessment"[All Fields] OR "assessment s"[All Fields] OR "assessments"[All Fields])) AND (("mental"[All Fields] OR "mentalities"[All Fields] OR "mentality"[All Fields] OR "mentalization"[MeSH Terms] OR "mentalization"[All Fields] OR "mentalizing"[All Fields] OR "mentalize"[All Fields] OR "mentalized"[All Fields] OR "mentally"[All Fields] OR ("psychiatrical"[All Fields] OR "psychiatrically"[All Fields] OR "psychiatrics"[All Fields] OR "psychiatry"[MeSH Terms] OR "psychiatry"[All Fields] OR "psychiatric"[All Fields]) OR ("psychologic"[All Fields] OR "psychological"[All Fields] OR "psychologically"[All Fields] OR "psychologization"[All Fields] OR "psychologized"[All Fields] OR "psychologizing"[All Fields]) OR "mental disorders"[MeSH Terms]) AND ("illness*"[All Fields] OR "disorder*"[All Fields] OR ("health"[MeSH Terms] OR "health"[All Fields] OR "health s"[All Fields] OR "healthful"[All Fields] OR "healthfulness"[All Fields] OR "healths"[All Fields]))) AND ("Ethiopia"[MeSH Terms] OR "Ethiopia"[All Fields] OR "Ethiopia s"[All Fields]) | 305 |
